# Hip pathology: the diagnostic accuracy of magnetic resonance imaging

**DOI:** 10.1186/s13018-018-0832-z

**Published:** 2018-05-29

**Authors:** Lucas Annabell, Vahid Master, Alexander Rhodes, Brett Moreira, Cassandra Coetzee, Phong Tran

**Affiliations:** 1 Department of Orthopaedics, Western Health, 160 Gordon Street, Footscray, Melbourne, VIC 3011 Australia; 2 Department of Radiology, Western Health, 160 Gordon Street, Footscray, Melbourne, VIC 3011 Australia

**Keywords:** Hip arthroscopy, Ligamentum teres, MRI, Magnetic resonance imaging

## Abstract

**Background:**

Hip arthroscopy has led to a greater understanding of intra-articular hip pathology. Non-contrast magnetic resonance imaging (MRI) is currently the gold standard in non-invasive imaging diagnosis, with high sensitivity in identifying labral pathology but equivocal results for ligamentum teres damage and chondral defects.

The aim of this study is to determine the accuracy of non-contrast MRI for diagnosis of intra-articular hip derangements and identify radiological features that could increase the accuracy of the diagnosis.

**Methods:**

A prospective study of 71 hips on 68 patients undergoing hip arthroscopy was conducted comparing pre-operative analysis of MRI imaging versus an arthroscopic examination. Two musculoskeletal radiologists reported the data independently. All hip arthroscopies were performed by a single surgeon. Patients with MRIs performed within 6 months before hip arthroscopy were included.

Outcome measures included observer accuracy identifying ligamentum teres tears, labral lesions, and chondral rim damage. Secondary outcome measures included inter-observer variability and correctly staged ligamentum teres tears.

**Results:**

The accuracy of radiology reporting for ligamentum teres tears, labral damage, and chondral rim lesions was 85.92% for each instance. The MRI findings most consistent with labral tears include the presence of linear high signal traversing the articular surface into the labrum, presence of intra-labral fluid signal, and loss of homogenous low signal triangular morphology. Chondral rim damage was difficult to diagnose, but abnormal signal at the chondrolabral junction with partial thickness defects would suggest damage. Ligamentum teres tears are commonly found but poorly graded. Thickening and increased signal suggests synovitis while discontinuity and fraying suggests partial tearing.

**Conclusion:**

Conventional non-arthrographic MRI offers an accurate non-invasive method to screen patients with symptoms referable to the hip by revealing the presence of labral tears, chondral defects, and ligamentum teres tears/synovitis. This study demonstrates that tears and synovitis of the ligamentum teres as potential sources of hip pain can be accurately identified on conventional non-arthrographic MRI. However, MRI has poor specificity and negative predictive value, and thus, a negative MRI result may warrant further investigation.

## Background

Hip pain in young adults can be a difficult clinical diagnosis. Common symptom generators include acetabular labral tears, ligamentum teres tears, and chondral damage. Conventional non-invasive imaging of the hip has a varied ability to detect lesions of the hip. Magnetic resonance imaging (MRI) has been shown to be more sensitive and specific than other non-invasive imaging techniques for the identification of ligamentum teres lesions [1, 2]. Magnetic resonance arthrography (MRA) has been shown to be more sensitive than conventional MRI but can be less specific and has recognised risks as an invasive procedure [3–5].

The acetabular labrum is a fibrocartilaginous ring that surrounds the bony acetabulum and blends inferiorly with the transverse acetabular ligament. It increases the joint surface area by adding depth to the acetabulum, and thereby reduces mechanical stress on the articular cartilage.

Younger individuals have a triangular labrum with sharply defined margins and homogeneous low signal that undergoes a progressive change in morphology, becoming rounded or blunted, and increasing in signal intensity with age. Although there is anatomic variation, the majority of changes seen in labral morphology are due to the dynamic and translational stresses placed on the hip labrum.

Minimal data is available for the diagnosis of ligamentum teres tears and chondral rim damage. It is also noted that it can be difficult to quantify the degree of damage with conventional imaging [6]. Furthermore, the experience of the radiologist reporting the images can affect the accuracy of diagnosis [7]. It has been shown that musculoskeletal-trained radiologists have better diagnostic accuracy than those without subspecialty training [8].

Arthroscopy has become a valuable diagnostic tool for hip joint pathology, as well as having a therapeutic capacity [9]. As with any surgery, it does have recognised complications. In patients where imaging has failed to provide a clear diagnosis however, direct visualisation of the joint by arthroscopy provides both diagnostic and therapeutic capacity.

Non-contrast MRI imaging is currently part of the pre diagnostic work up of hip pain at Western Health, Victoria. This study was designed to analyse the sensitivity and specificity of MRI in identifying intra-articular hip pathology, specifically, tears of the ligamentum teres and chondral rim damage, in comparison to hip arthroscopy at a single institution.

## Methods

A retrospective review of a consecutive series of hip arthroscopies performed between March 2011 and January 2013 was conducted. A total of 71 cases (41 male, 30 female) with MRI in 68 patients were thus available for prospective review by musculoskeletal radiologists.

### MRI

The 71 cases were obtained from four different radiology providers. All had been previously reported by board-certified radiologists. These images were re-reported by two musculoskeletal-trained radiologists blinded to the original report and the findings at hip arthroscopy.

The MRI sequences reviewed included coronal T1, coronal PD FS, sagittal PD FS, and Ax Obl PD FS sequences. All scans were performed on a GE 3 Tesla twin speed HDx (Peak gradient 790 gauss/cm). All studies were judged to be of diagnostic quality.

### Hip arthroscopy

All hip arthroscopies were performed by a single surgeon between March 2011 and January 2013. All patients were intraoperatively reviewed for labral tears, ligamentum teres tears, and chondral rim damage. All operative reports were available and collected for review for the purposes of this study.

### Comparison of findings

**Fig. 1:**
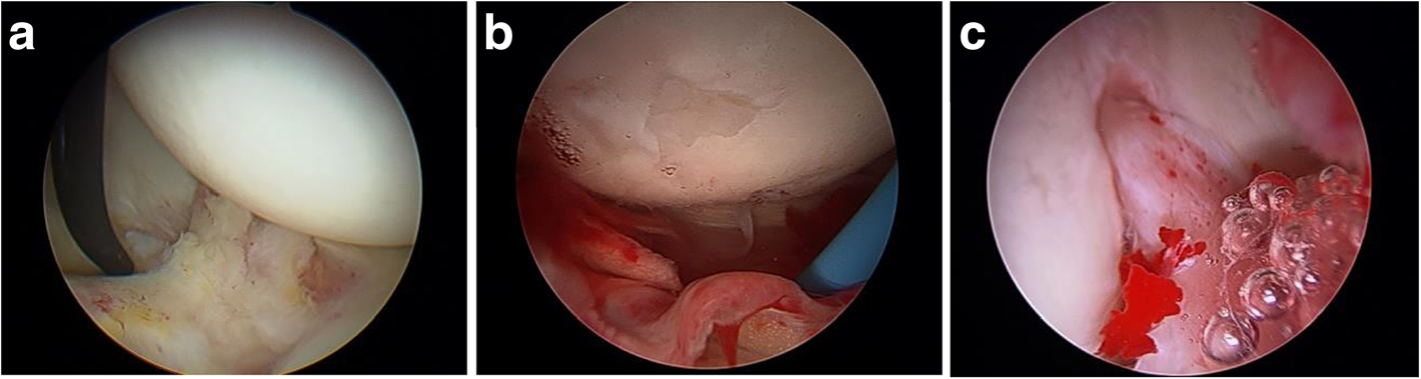
Arthroscopic findings. **a** Labral tear. **b** Ligamentum teres tear and chondral damage. **c** Inflamed ligamentum teres

**Fig. 2:**
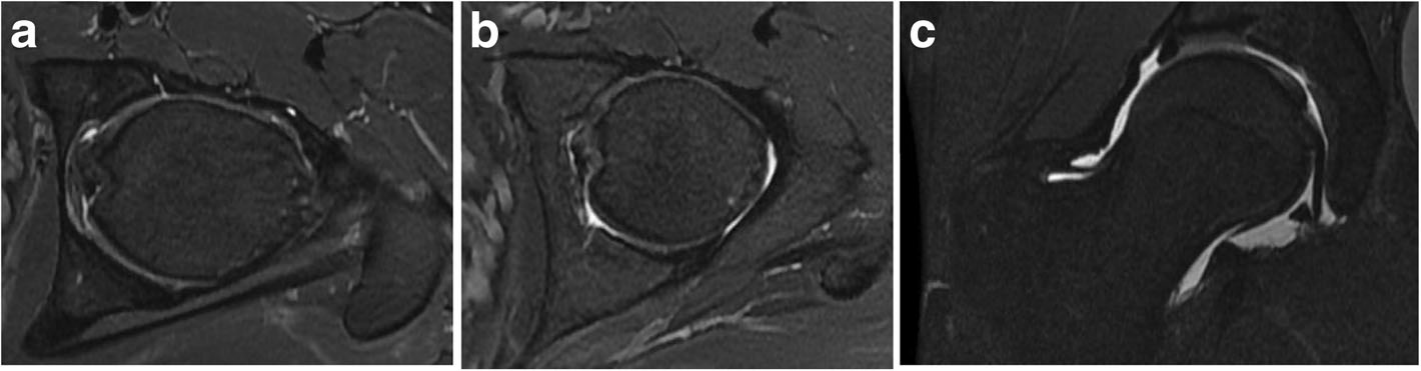
MRI findings. **a** Chondral rim damage. **b** Ligamentum teres tear. **c** Labral tear

The MRI reports and arthroscopic findings were compared for the investigation of labral tears, ligamentum teres damage, and chondral rim damage. For the arthroscopic findings, labral tears were graded in direction and magnitude. Ligamentum teres tears were graded using the Gray and Villar classification (Table 1) [10]. Chondral rim damage was graded using a modified Outerbridge classification (Table 2). Both the acetabular and femoral articular surfaces were assessed. The articular cartilage was graded on the MRI and at arthroscopy the classification system of Outerbridge. Grade 0 indicated intact articular cartilage; grade 1, chondral softening (high signal); grade 2, superficial ulceration, fissuring, or fibrillation involving less than 50% of the depth of the articular surface; grade 3, ulceration, fissuring, or fibrillation involving more than 50% of the depth of the articular cartilage; and grade 4, full-thickness chondral wear with exposure of subchondral bone. All chondral lesions on MRI were confirmed in at least two separate planes and specific attention to the weight-bearing acetabulum, fovea, and posterior joint space. Figure 1 shows examples of some of the pathology noted on arthroscopy while Fig. 2 shows pathology reported by the radiologists.

**Table 1:** Grading system for ligamentum teres tears

Grade of tear	Modified Gray and Villar classification
0	No tear
1	Low grade tear < 50%
2	High grade tear > 50%
3	Complete tear

**Table 2:** Grading system for chondral rim damage

Grade of chondrosis	Original Outerbridge classification	Modified Outerbridge classification for acetabular chondrosis
1	Softening and swelling of cartilage	Softening of cartilage
2	Fragmentation and fissuring in an area 1.5 cm or less in diameter	Cleavage tear
3	Fragmentation and fissuring to the level of subchondral bone in an area with a diameter more than 1.5 cm	Delamination
4	Exposed subchondral bone	Erosion of cartilage down to exposed bone

The radiologists were asked to report whether damage was present, and also to what grade, for each of the three pathologies. Each radiologist’s reports were also compared to investigate the inter-observer reliability of the findings.

### Statistics

The accuracy of the MRI reports were judged by the arthroscopic findings and were calculated by true positives, true negatives, false positives, and false negatives. From these results, positive predictive values, negative predictive values, overall sensitivity, specificity, and accuracy were calculated.

Statistical analysis was performed with SPSS software. Paired student *T* tests were conducted to determine the accuracy of each observer. All *p* values were given a statistical significance value of 0.05. Inter-observer reliability scores were calculated with kappa values. Scores between 0 and 0.20 were graded as slight, 0.21 and 0.40 as poor, 0.41 and 0.60 as fair, 0.61 and 0.80 as moderate, and 0.81 and 1 as substantial.

## Results

There were 71 patients, with an age range of 21 to 66, and an average age of 39. There were 30 women (42%) and 41 men (58%). There were 35 right hips and 38 left hips examined. The average time between MRI and hip arthroscopy was 71 days (range 1 to 179 days).

### Ligamentum teres tears

At arthroscopy, 63 patients had a grade 1, 2, or 3 tear of their ligamentum teres. Observer A correctly identified 55 out of 63 tears. From the 8 cases that were normal at arthroscopy, observer A correctly reported 2 while he described 6 as torn. Observer B correctly identified 59 out of 63 tears, and from the 8 normal ligamentum teres cases, he described 2 as torn and 6 correctly. Table 3 outlines the results of the individual observers comparing MRI results with arthroscopic findings. Table 4 outlines the average accuracy between observers A and B. The accuracy between observers A and B was taken as the mean of the individual observer results.

**Table 3:** MRI results compared with arthroscopy—individual observers

	Accuracy		Sensitivity (%)		Specificity (%)		PPV (%)		NPV (%)	
Observer	A	B	A	B	A	B	A	B	A	B
Lig Teres	80.28	91.55	88.71	93.65	22.22	75.00	88.71	96.72	22.22	60.00
95% CI	–	–	78.11–95.34	84.53–98.24	2.81–60.01	34.91–96.81	78.11–95.34	88.55–99.61	2.81–60.01	26.24–87.84
Labrum	90.14	81.69	96.43	83.93	66.67	73.33	91.53	92.16	83.33	55.00
95% CI	–	–	87.69–99.56	71.67–92.38	38.38–88.18	44.9–92.21	81.32–97.19	81.12–97.82	51.59–97.91	31.53–76.94
Chondral rim	85.92	85.92	93.22	89.47	50.00	71.43	90.16	92.73	60.00	62.50
95% CI	–	–	83.54–98.12	78.48–96.04	21.09–78.91	41.9–91.61	79.70–96.34	82.41–97.98	26.24–87.84	34.52–85.41

*Abbreviations*: *PPV* positive predictive value, *NPV* negative predictive value

**Table 4:** MRI results compared with arthroscopy—combined results

	Accuracy	Sensitivity (%)	Specificity (%)	PPV (%)	NPV (%)
Lig Teres	85.92	91.20	47.06	92.68	42.11
95% CI	–	84.80–95.52	22.98–72.19	86.56–96.6	19.72–67.17
Labrum	85.92	90.18	70.00	91.82	65.63
95% CI	–	83.11–94.99	50.60–85.27	85.00–96.21	46.81–81.43
Chondral rim	85.92	91.38	61.54	91.38	61.54
95% CI	–	84.72–95.79	40.57–79.77	84.72–95.79	40.13–80.10

*Abbreviations*: *PPV* positive predictive value, *NPV* negative predictive value

### Labral tears

For the patients who had labral tears identified at arthroscopy, observer A correctly identified 54 out of 56 cases while observer B correctly identified 47 cases. Of the 15 cases without tears, observer A reported 10 correctly and 5 incorrectly. Observer B reported 11 correctly and 4 incorrectly.

### Chondral damage

The comparison of MRI grading with arthroscopy within 1 grade was correct in 54 out of 58 cases with chondral damage for observer A, while observer B identified 51 positive cases correctly. For the 13 patients with no chondral disease at arthroscopy, observer A incorrectly reported 6 cases and correctly reported 7 while observer B incorrectly reported 4 cases and correctly reported 9 cases.

### Inter-observer variation

Of the cases positive for pathology, inter-observer variation for ligamentum teres tears, the kappa value was 0.470 (fair); for labral tears, the kappa value was 0.501 (fair); and for chondral damage, the kappa value was 0.510 (fair).

## Discussion

Hip arthroscopy and direct visualisation of the hip joint is the gold standard for the diagnosis of internal derangement of the hip joint, with X-ray, ultrasound, computed tomography (CT), and bone scan providing very little information regarding the internal structures of the hip joint.

MRA with fluoroscopically guided intra-articular contrast (Gadolinium) injection has shown the best correlative results after hip joint arthroscopy, for labral tears with 92% sensitivity and 100% specificity [11]. Chondral rim derangement detection is, however, poor, demonstrating 79% sensitivity and 77% specificity. Concomitant ligamentum teres tear and synovitis detection is limited, demonstrating 1.8% sensitivity and 98.5% specificity [12].

Furthermore, all invasive techniques, including MRA with intra-articular contrast injection, are associated with a risk of infection and pain. MRI without intra-articular contrast injection is the non-invasive imaging of choice for the hip, demonstrating lower risk of infection and pain, being readily available and being of lower cost.

In the current literature, non-contrast MRI is moderately accurate for identifying labral tears (85% sensitivity) and chondral rim damage (92% sensitivity) [13]. MRI is poor at quantifying chondral damage [14].

3 Tesla MRI has also been confirmed as superior to 1.5 Tesla for quantifying internal derangement of the hip [15].

Our results demonstrated reasonable sensitivity (90.92%) and positive predictive values (91.96%). The intra-observer reliability was only fair, and this may be attributed to a learning effect of each observer as the study progressed. We found MRI to be poorly specific and inaccurate for measuring or grading chondral lesions. MRI was shown to have a poor negative predictive value. The investigators did note that there was an average time difference of 71 days between patients undergoing the MRI and arthroscopy. This difference in time may have resulted in deterioration of the lesions between having the MRI and undergoing the arthroscopy; leading to larger- more detectable- lesions at the time of arthroscopy.

Assessment for ligamentum teres damage was based on alteration in the normal smooth contour and loss of the normal low signal. Focal high signal with loss of the normal smooth contour was indicative of a partial ligamentum teres tear, while complete loss of continuity indicated complete tear. High signal change within and surrounding the ligamentum was considered indicative of synovitis. Changes in signal intensity reflect degeneration and injury, simulating the changes seen in the meniscal fibrocartilage of the knee. The labrum is subject to continuous weight-bearing stresses, undergoing morphologic change, and degeneration with age [16]. Abnormal hip stress results in labral injury, which usually manifests as a tear, sometimes with displacement or detachment of a labral flap [17].

The MRI criteria used for a labral tear was a line of high signal coursing from the articular side through the base or into the substance of the labrum, with or without distraction of the labrum [18]. Chondrolabral junction signal abnormality was regarded as a labral tear, although this may be difficult to distinguish from an articular cartilage abnormality at the base of the labrum.

Importantly, chondral damage grading is poorly accurate with the planar sequences used, the cartilage only well seen (directly perpendicular) on two to three slices on the coronal PDFS sequences; therefore, the whole cartilage surface was not seen in its entirety on conventional imaging.

The limitations of this study included possible sampling bias, with the high prevalence of disease in the patient cohort, which clearly reflects the presentation of hip symptoms. Patients without hip pain were not included.

## Conclusion

This study demonstrates that tears and synovitis of the ligamentum teres, as potential sources of hip pain, can be accurately identified on conventional non-arthrographic MRI. MRI is able to detect the presence of labral tears, chondral defects, and ligamentum teres tears/synovitis; however, MRI was found to be poor at grading the pathology compared to direct visualisation during arthroscopy. MRI has poor specificity and negative predictive value, and thus, a negative MRI result may warrant further investigation. Conventional non-arthrographic MRI offers an accurate non-invasive method to screen patients with symptoms referable to the hip.

This study also confirmed a relative lack of accuracy with respect to the grading of cartilage abnormalities, largely due to the narrow zone of visualisation.

## Abbreviations

CI: Confidence interval
CT: Computed tomography
MRA: Magnetic resonance arthrography
MRI: Magnetic resonance imaging
NPV: Negative predictive value
PPV: Positive predictive value
